# Widely Used Herpes Simplex Virus 1 ICP0 Deletion Mutant Strain *dl*1403 and Its Derivative Viruses Do Not Express Glycoprotein C Due to a Secondary Mutation in the gC Gene

**DOI:** 10.1371/journal.pone.0131129

**Published:** 2015-07-17

**Authors:** Cristina W. Cunha, Kathryne E. Taylor, Suzanne M. Pritchard, Mark G. Delboy, Tri Komala Sari, Hector C. Aguilar, Karen L. Mossman, Anthony V. Nicola

**Affiliations:** 1 Department of Veterinary Microbiology and Pathology, College of Veterinary Medicine, Washington State University, Pullman, Washington, United States of America; 2 Department of Pathology and Molecular Medicine, McMaster Immunology Research Centre, Institute for Infectious Disease Research, McMaster University, Hamilton, Ontario, Canada; 3 Department of Microbiology and Immunology, Virginia Commonwealth University School of Medicine, Richmond, Virginia, United States of America; 4 Paul G. Allen School for Global Animal Health, Washington State University, Pullman, Washington, United States of America; University of Alberta, CANADA

## Abstract

Herpes simplex virus 1 (HSV-1) ICP0 is a multi-functional phosphoprotein expressed with immediate early kinetics. An ICP0 deletion mutant, HSV-1 *dl*1403, has been widely used to study the roles of ICP0 in the HSV-1 replication cycle including gene expression, latency, entry and assembly. We show that HSV-1 *dl*1403 virions lack detectable levels of envelope protein gC, and that gC is not synthesized in infected cells. Sequencing of the gC gene from HSV-1 *dl*1403 revealed a single amino acid deletion that results in a frameshift mutation. The HSV-1 *dl*1403 gC gene is predicted to encode a polypeptide consisting of the original 62 N-terminal amino acids of the gC protein followed by 112 irrelevant, non-gC residues. The mutation was also present in a rescuant virus and in two *dl*1403-derived viruses, D8 and FXE, but absent from the parental 17+, suggesting that the mutation was introduced during the construction of the *dl*1403 virus, and not as a result of passage in culture.

## Introduction

Herpes simplex virus 1 (HSV-1) is a prototype virus of the *Alphaherpesvirinae* subfamily that causes lifelong latent infections in humans. Upon infection of the host cell, HSV-1, like all herpesviruses, executes a cascade of temporally regulated gene expression. Infected cell protein (ICP0) is an HSV-1 immediate early (IE) phosphoprotein that acts as a promiscuous transactivator of viral and cellular genes, and is required for low multiplicity infection [1,2,3,4,5]. ICP0 is important for progression to lytic infection and for reactivation from latency [6,7,8,9]. Although initially thought to function in the nucleus by targeting repressive cellular proteins for degradation using the E3 ubiquitin ligase activity of its RING finger domain (reviewed in [1], ICP0 has been more recently suggested to have additional functions in the cytoplasm [10,11,12], and many of its binding partners are not directed to the proteasome [13,14,15,16,17,18,19,20,21,22]. In addition to being expressed in the host cell, ICP0 is a minor structural component of the tegument layer of viral particles [23,24,25,26,27,28,29]. Tegument ICP0 has been proposed to regulate transport of entering viral capsids to the nuclear pore complex in a proteasome-dependent manner [30,31].

HSV-1 *dl*1403 is an ICP0 deletion mutant virus derived from wild type strain 17+. The ICP0 gene is present in two copies within the HSV-1 genome, one each in the TR_L_ and IR_L_ inverted repeat regions. Bearing a 2 kilobase deletion in both copies of the ICP0 gene, HSV-1 *dl*1403 was constructed via homologous recombination between the HSV-1 17+ genome and a plasmid specifying the ICP0 gene containing a 2 kb deletion [4]. The resultant virus has been used in many studies of ICP0 function [30,32,33,34,35,36,37,38,39]. Here, we demonstrate that HSV-1 *dl*1403 contains a previously unrecognized secondary mutation that renders it incapable of synthesizing the wild type gC gene product.

## Materials and Methods

### Cells and viruses

Vero, HEL and U2OS cells (American Type Culture Collection, Rockville, MD) were propagated in Dulbecco modified Eagle medium (Invitrogen, Grand Island, NY) supplemented with 10% fetal bovine serum (Gemini Bio-Products, West Sacramento, CA). CHO-nectin-1 (M3A) cells (provided by Roselyn Eisenberg and Gary Cohen, University of Pennsylvania) are stably transformed with the nectin-1 gene and contain the *E*. *coli lacZ* gene under the control of the HSV-1 ICP4 promoter. The cells were propagated in Ham F-12 nutrient mixture (Invitrogen) supplemented with 10% fetal bovine serum, 150 ug of puromycin (Sigma, St. Louis, MO)/ml, and 250 ug of G418 sulfate (Fisher Scientific, Fair Lawn, NJ)/ml.

HSV-1 wild-type Glasgow strain 17 syn^+^ (17+) [40], its ICP0 mutant derivative *dl*1403, the rescuant *dl*1403R, and *dl*1403-derived mutants FXE and D8 were provided by Roger Everett, MRC Virology Unit, Glasgow, United Kingdom. HSV-1 *dl*1403 has a 2 kilobase lesion in both copies of the ICP0 gene [4]. FXE, D8 and the rescued virus *dl*1403R were obtained by co-transfection of mutant virion DNA and a plasmid containing a fragment specifying the ICP0 gene [41,42]. Wild-type HSV-1 strain KOS and its derivative 7134, which contains the *lacZ* gene in place of both inverted repeat copies of the ICP0 gene [43] and the KOS-derived ICP0-null virus n212 [44] were obtained from P. Schaffer (Harvard University). HSV-1 KOS-tk12 contains the *lacZ* gene under the control of the viral ICP4 promoter [45] and was obtained from P. Spear (Northwestern University). The ICP0-null virus 7910 derived from HSV-1 strain F was obtained from B. Roizman (University of Chicago). HSV-1 KOS-derived mutant gC∆2–3 (provided by Curtis Brandt, University of Wisconsin) lacks gC coding sequences [46]. 17+, *dl*1403, *dl*1403R, FXE, D8, n212, 7910 and 7134 virus stocks were grown and titered on U2OS cells. KOS and gC∆2–3 virus stocks were grown and titered on Vero cells.

### Antibodies

Mouse monoclonal antibody H1A027 (Virusys, North Berwick, ME) recognizes ICP0. R47 is a rabbit polyclonal antibody to gC [47], and DL6 is a mouse MAb to gD [48] (both provided by Gary Cohen and Roselyn Eisenberg). Mouse MAb H1817 (Virusys) recognizes gB, and mouse MAb AC-74 (Sigma) recognizes beta-actin.

### SDS-PAGE and Western blot analysis

Samples in Laemmli buffer were separated by SDS polyacrylamide gel (4–20% gradient) electrophoresis. Gels were either fixed and stained with Coomassie blue (Sigma) or blotted onto nitrocellulose and probed with 1 μg of mouse monoclonal antibody (MAb)/ml specific for HSV gB, VP5 (MAbs H1359, H1A021, respectively, Santa Cruz), ICP0 (MAb 11060, Virusys, Sykesville, MD), or 0.01 μg MAb 1–21 to VP16 (Virusys). Nitrocellulose membranes were incubated with horseradish peroxidase-conjugated goat anti-mouse immunoglobulin G (Pierce, Rockford, IL), developed with enhanced chemiluminescence detection reagents (Pierce), and exposed to X-ray film (Kodak) [49].

### DNA sequencing

DNA sequence from HSV-1 17+, *dl*1403, and *dl*1403R viruses was amplified by PCR using the forward primer 5’ GAGGGGGAGGCGTCGG (this study) and reverse primer 5’ CGGACGACGTACACGATT [50]. PCR products were electrophoresed on a 1% agarose gel, and the 1520 bp band corresponding to the gC gene was cut from the gel. DNA was purified from gel using a MiniElute PCR purification kit (Qiagen) and sequenced with the PCR primers. Sequences were analyzed with the Vector NTI Advance (Life Technologies).

### RT-PCR

Total RNA was extracted from Vero cells infected with HSV-1 17+ or *dl*1403 (MOI of 1) for 24 hours using the iPrep TRIzol Plus RNA kit per the manufacturer's instructions (Life Technologies), modified to include DNAse treatment. RNA was converted into cDNA using the iScript Advanced cDNA synthesis kit (Bio Rad). gC transcripts was detected using the CFX96 Real-Time PCR Detection System (Bio-Rad) and forward primer 5’GTCCACCCTGCCCATTTC (this work) and reverse primer 5’ CGGACGACGTACACGATT [50].

### Effect of proteasome-inhibitor MG132 on HSV entry

Confluent CHO-nectin-1 cell monolayers grown in 96-well dishes were treated with culture medium containing MG132 for 15 min at 37°C. HSV-1 KOS, 7134, gC∆2–3, 17+ or *dl*1403 (multiplicity of infection [MOI] of 1) was added. Cells were incubated in the constant presence of agent for 7 h. 0.5% Nonidet P-40 (Sigma) cell lysates were prepared, chlorophenol red-beta-D-galactopyranoside (Roche Diagnostic, Indianapolis, IN) was added, and the beta-galactosidase activity was read at 595 nm with an ELx808 microtiter plate reader (BioTek Instruments, Winooski, VT). The MG132 treatments tested had no adverse effect on cell viability as measured by trypan blue exclusion [31]. Beta-galactosidase activity indicated successful entry [51]. Mean results and standard errors were calculated for four replicate samples.

### Effect of heparin on HSV-1 infectivity

Confluent Vero cells were pre-chilled on ice. HSV-1 17+, *dl*1403, KOS, or gC∆2–3 (100 PFU per well) was mixed with indicated heparin concentrations in carbonate-free DMEM containing 5 mM HEPES and 0.2% BSA. Chilled inocula were added to cells and incubated at 4˚C on ice for 1 hr to allow virus binding to the cell surface. Cultures were washed thrice with PBS, and then incubated at 37˚C for 24 hr. Plaque formation was detected by immunoperoxidase staining with anti-HSV-1 polyclonal antibody HR50 (Fitzgerald Industries) [52]. Plaque formation in the untreated sample was set to 100%. The data are means of quadruplicate determinations with the standard error.

### Viral growth assays

HEL cells were infected with the indicated viruses for 24 hr (MOI of 10). Cells and supernatant media were harvested, freeze-thawed three times, and then titered on U2OS cells in the presence of hexamethylene bisacetamide (HMBA) and 2% human serum. After three days, cells were fixed with methanol, stained with Giemsa (Sigma), and then plaques were counted.

## Results and Discussion

### HSV-1 *dl*1403 virions lack gC protein

**Fig 1:**
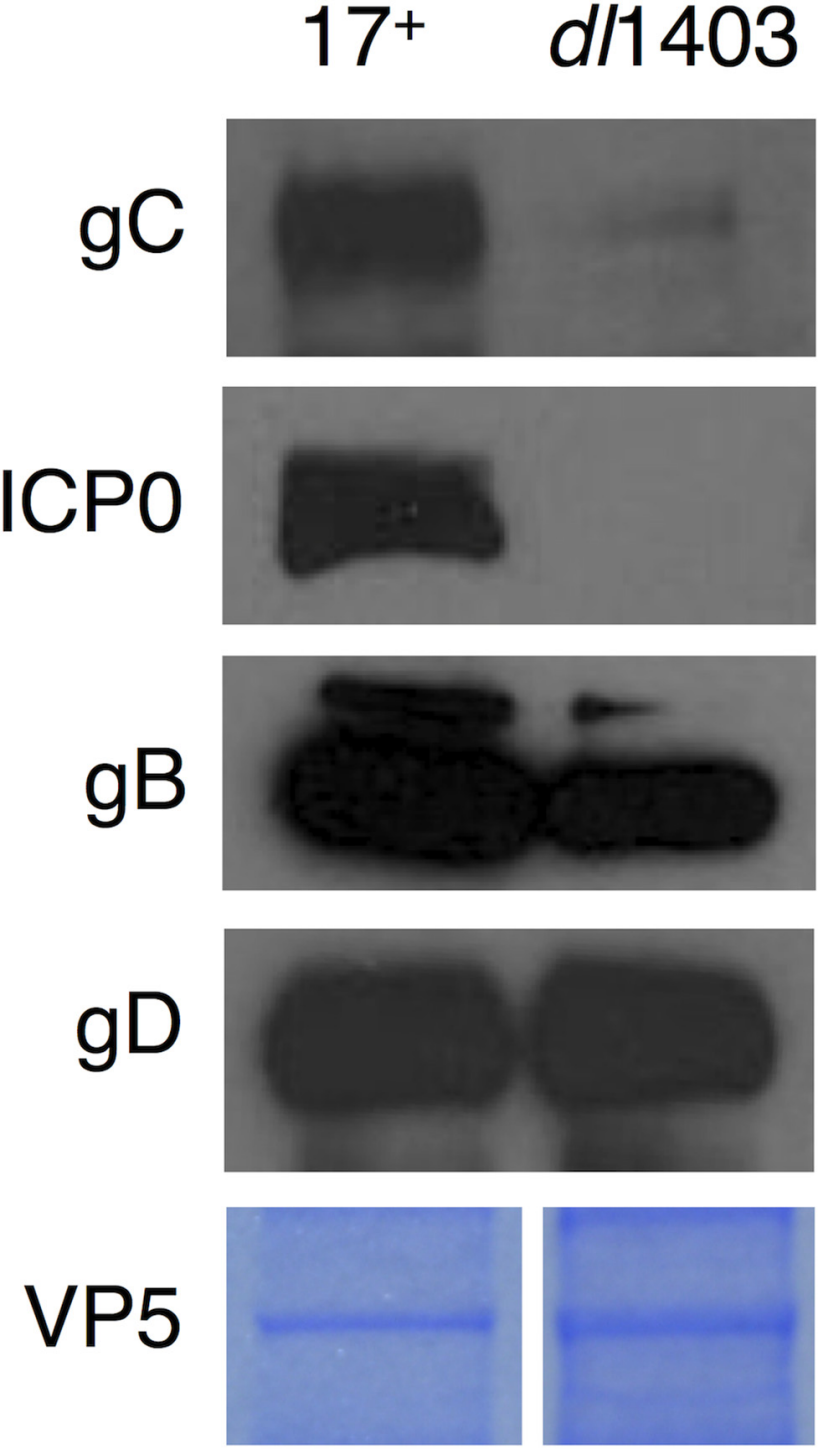
Virion gC is undetectable in the HSV-1 ICP0 deletion mutant, *dl*1403. Equivalent VP5 units of extracellular 17+ or *dl*1403 virions were analyzed by SDS-PAGE followed by Western blotting with antibodies against the indicated proteins. VP5 content of virion preparations was determined by SDS-PAGE and Coomassie staining.

ICP0-null virions have a protein composition similar to that of the wild type virions. Specifically, the HSV-1 proteins VP5, VP1/2, ICP4, VP16, VP22, VP13/14, gB, gD, gH, and gL are incorporated into extracellular *dl*1403 virions in the absence of ICP0 [24]. To continue this line of inquiry, equivalent VP5 units of wild type 17+ virions or *dl*1403 virions were analyzed by SDS-PAGE and Western blotting with polyclonal antibody to gC. Interestingly, gC was not detectable in *dl*1403 virions (Fig 1). As expected, tegument ICP0 was also not detected. Virion gB and gD did not appear to be reduced in the absence of ICP0 (Fig 1) in agreement with previous observations [24].

**Fig 2:**
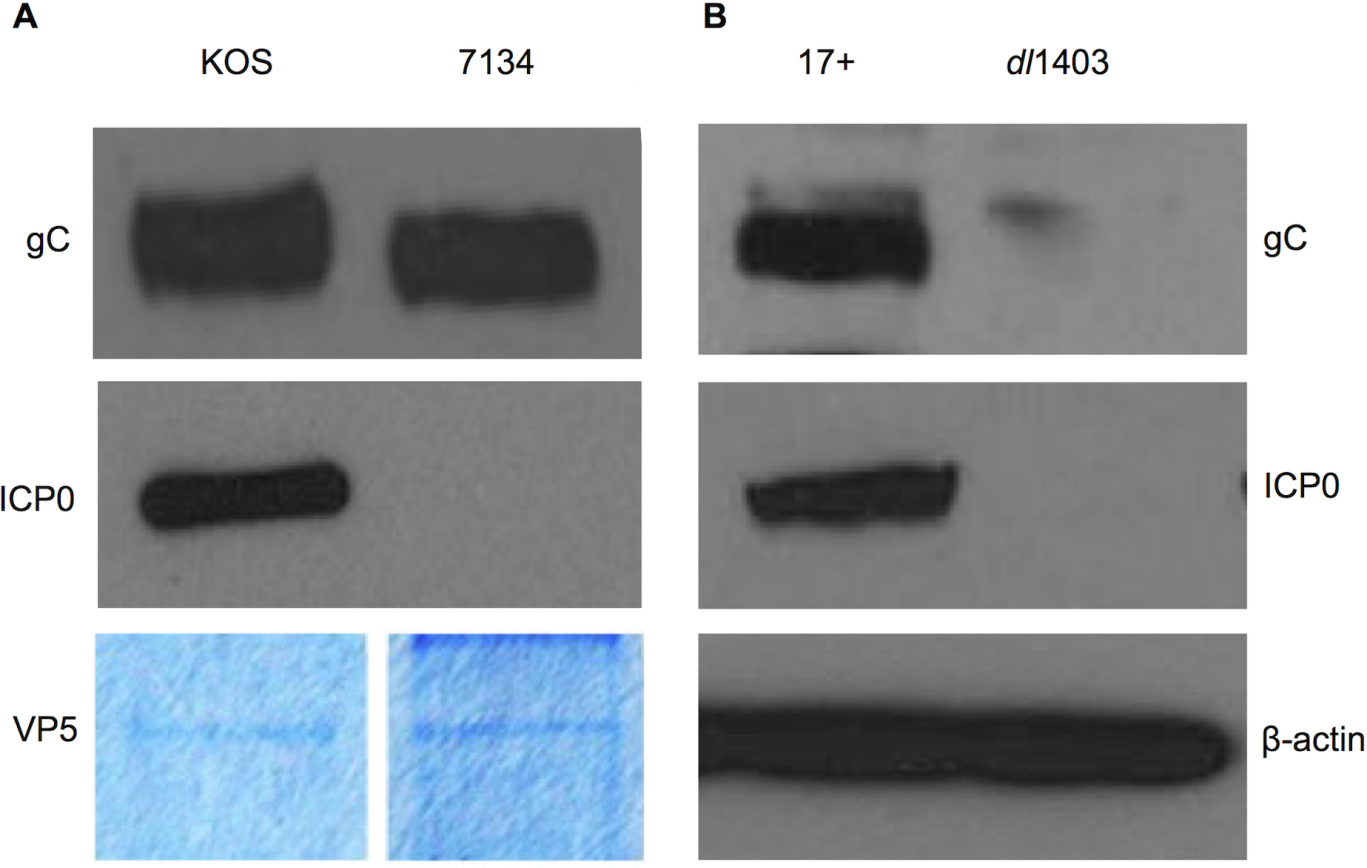
Detection of gC in cells infected with HSV-1 *dl*1403. **A.** U2OS cells were infected with the indicated virus (MOI of 1) for 18 h. Cell lysates were analyzed by SDS-PAGE followed by Western blotting with PAb R47 to gC, MAb H1A027 to ICP0, or MAb AC-74 to beta-actin (Sigma). **B.** Equivalent VP5 units of extracellular HSV-1 KOS or 7134 virions were analyzed by SDS-PAGE followed by Western blotting with antibodies against the indicated proteins. VP5 content of virion preparations was determined by SDS-PAGE and Coomassie staining.

To address one possible reason for the absence of gC from *dl*1403 virions, we determined whether gC was present in the viral particles of a different ICP0-null HSV-1. We utilized the ICP0-null virus, HSV-1 7134, which was constructed in an HSV-1 wild type KOS background. gC was detected in 7134 virions (Fig 2B), suggesting that gC can be assembled into viral particles in the absence of ICP0.

### gC is not detectable in cells infected with HSV-1 *dl*1403

We next determined whether *dl*1403-infected cells expressed gC. HSV-1 *dl*1403-infected cell lysates were analyzed by SDS-PAGE and Western blot. There was no detectable gC (or ICP0) present in the *dl*1403-infected cells (Fig 2A). Lysates of the wild type 17+-infected cells contained detectable levels of gC and ICP0 (Fig 2A). gC was also readily detected in cells infected with ICP0-null viruses n212, 7134 (KOS strain) and 7910 (F strain) that were generated independently from *dl*1403 (data not shown). Thus, the results suggest that gC was undetectable in *dl*1403 virions (Fig 1) because gC protein was not expressed in the infected cells from whence they came. Similarly, cells infected with the *dl*1403-derived viruses, D8, in which the nuclear localization signal of ICP0 has been disrupted, and FXE, which lacks the RING finger domain of ICP0, also lacked detectable gC (data not shown). Therefore, the possibility that HSV-1 *dl*1403 contained a previously unrecognized mutation in its gC gene was investigated.

### HSV-1 *dl*1403 contains a frameshift mutation in its gC gene

**Fig 3:**
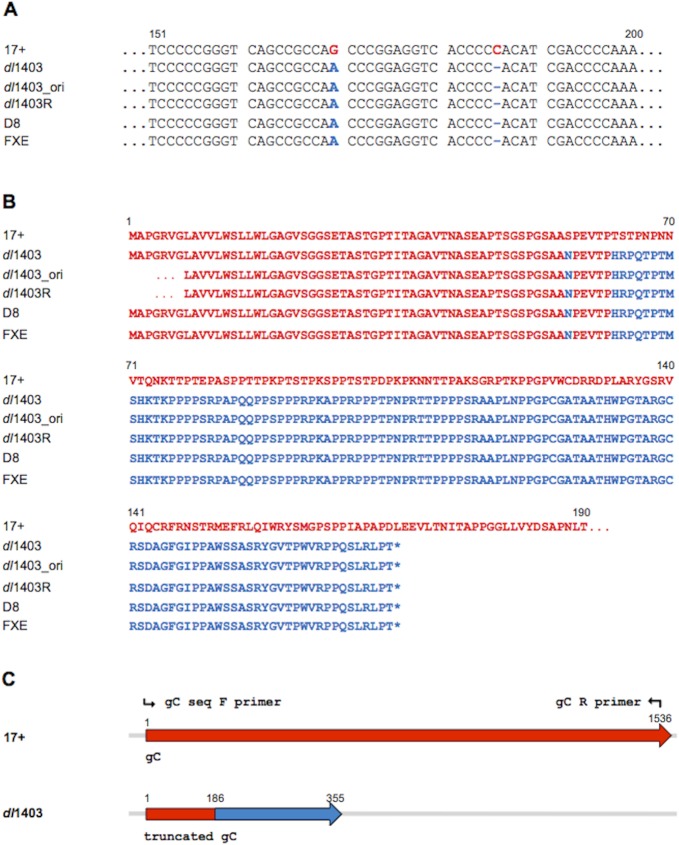
Sequence analysis of the HSV-1 gC gene from parental wild type 17+ and *dl*1403 derivatives. **A.** Nucleotide alignment of a fragment of the gC gene from the current passage of HSV-1 *dl*1403 in our laboratory (*dl*1403), from the original *dl*1403 stock received by our lab (*dl*1403_ori), from the original HSV-1 *dl*1403R (ICP0 rescuant) received by our lab as well as from D8 and FXE, ICP0 mutant viruses derived from *dl*1403. Nucleotide differences are indicated in color between wild type 17+ (blue) and the virus derivatives (red). Deletion is indicated by "-". Numbering of nucleotides is indicated at the top. **B.** Alignment of gC proteins from the four virus preparations based on DNA sequence results. Sequences in gray have no homology to the gC protein expressed by the parental HSV-1 17+. Stop codons are indicated by “*”. Numbering of amino acids is indicated in the top. **C.** Schematic representation of the region of the genomes of HSV-1 17+ and *dl*1403 encoding the gC gene. The DNA sequence encoding gC is in black, and the frameshifted coding sequence that results in altered protein sequence is in gray. Forward and reverse primers used for sequencing are indicated.

Sequencing the gC gene from HSV-1 *dl*1403 revealed a single nucleotide deletion of C186 relative to the wild type parent 17+, which results in a frameshift (Fig 3A). The new reading frame introduces a premature stop codon at nucleotide positions 356–358. Unlike the wild type gC polypeptide which is 511 amino acids, the predicted polypeptide encoded by the *dl*1403 gC gene consists of the first 62 native gC residues followed by 112 non-gC amino acids (Fig 3B and 3C). The sequence also revealed a G to A substitution at nucleotide 170 (Fig 3A), which corresponds to a predicted S56N amino acid mutation (Fig 3B). The *dl*1403 virus that was sequenced was the same passage that was analyzed in Figs 1 and 2. Identical sequencing results were obtained with the earliest passage virus to which our two laboratories had access (*dl*1403_ori). Thus, in addition to not expressing ICP0, HSV-1 *dl*1403 fails to express a detectable gC protein likely due to these mutations.

To explore further whether the mutations in the gC gene arose during recent passage in cell culture, we sequenced the gC gene from *dl*1403R, a *dl*1403 virus that was rescued with the wild type ICP0 gene, and from FXE and D8, ICP0 mutants derived from *dl*1403. The gC gene from each of these *dl*1403-derived viruses contained the same substitution and frameshift mutations that were detected in all preparations of *dl*1403 tested (Fig 3A). These results suggest that the gC mutations were introduced during the original construction of the *dl*1403 virus.

Despite the detected mutations in the gC gene, we addressed the formal possibility that HSV-1 *dl*1403 may lack the gC protein due to an inability to synthesize gC mRNA. gC transcripts containing a region downstream of the substitution and frameshift mutations were detected by RT-PCR in both *dl*1403-infected cells and wild type infected cells (data not shown). Together, the results suggest that *dl*1403 virions and infected cells lack detectable levels of gC, due to the identified mutations in the *dl*1403 gC gene.

### Entry of ICP0-null mutants is resistant to inhibition by MG132, regardless of the presence of gC

**Fig 4:**
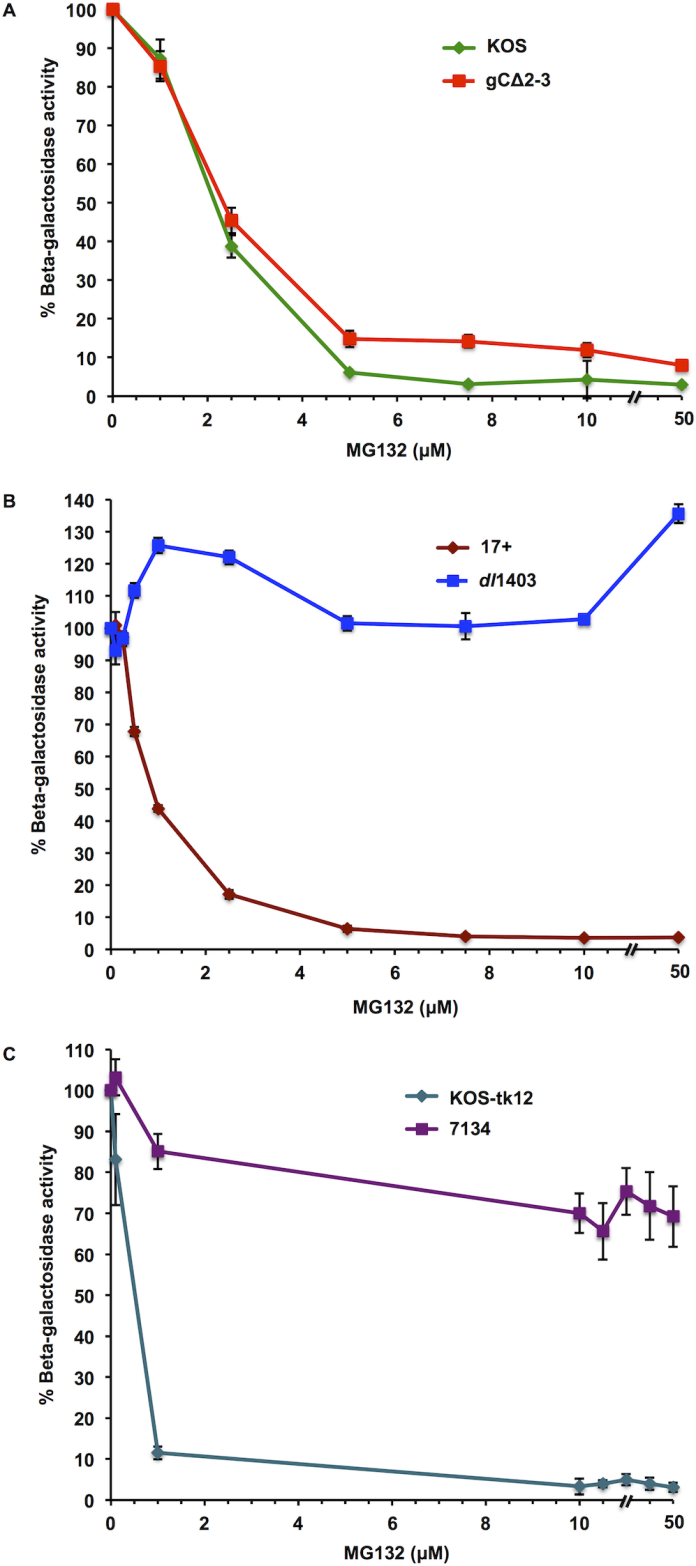
Effect of proteasome inhibitor MG132 on the entry of HSV-1 that lacks gC. CHO-nectin-1 cells (**A, B**) or Vero cells (**C**) were treated with MG132 for 15 min at 37°C. HSV-1 KOS, gC∆2–3, 17+, *dl*1403, KOS-tk12, or 7134 was added (MOI of ~1) for 6 h. The percent beta-galactosidase activity relative to that obtained in the absence of MG132 is indicated. The data are means of quadruplicate determinations with the standard error of the mean. Results are representative of three independent experiments.

ICP0 present in the virion tegument layer regulates the proteasome-dependent delivery of incoming viral capsids to the nuclear pore complex [30]. The entry of ICP0-null virions is less sensitive to inhibition by proteasome inhibitors. Specifically, we showed previously that the entry of wild type HSV-1 strain 17+ was inhibited by the proteasome inhibitor MG132, a peptide aldehyde, in a concentration dependent manner, but HSV-1 *dl*1403 was refractory to inhibition [30]. Since *dl*1403 lacks gC in addition to ICP0, we assessed directly whether gC contributes to the proteasome-dependence of HSV-1 entry. The effect of MG132 on the entry of a gC-null (ICP0^+^) virus, HSV-1 gC∆2–3, was determined. MG132 inhibited the entry of gC∆2–3 in a concentration-dependent manner as measured by beta-galactosidase reporter gene expression, similar to the wild type virus (KOS) from which it was derived (Fig 4A). The highest concentration of MG132 inhibited > 90% of entry of either virus. These results suggest that gC does not contribute to the reliance of HSV-1 entry on the degradative activity of the proteasome. In contrast, as demonstrated previously [29,30], the entry of viruses that lacked gC and ICP0 (*dl*1403; Fig 4B) or lacked ICP0 alone (7134; Fig 4C), were refractory to MG132 relative to matched viruses that contained ICP0.

### HSV-1 *dl*1403 is more resistant to inhibition by heparin than is its HSV-1 17+ parent

**Fig 5:**
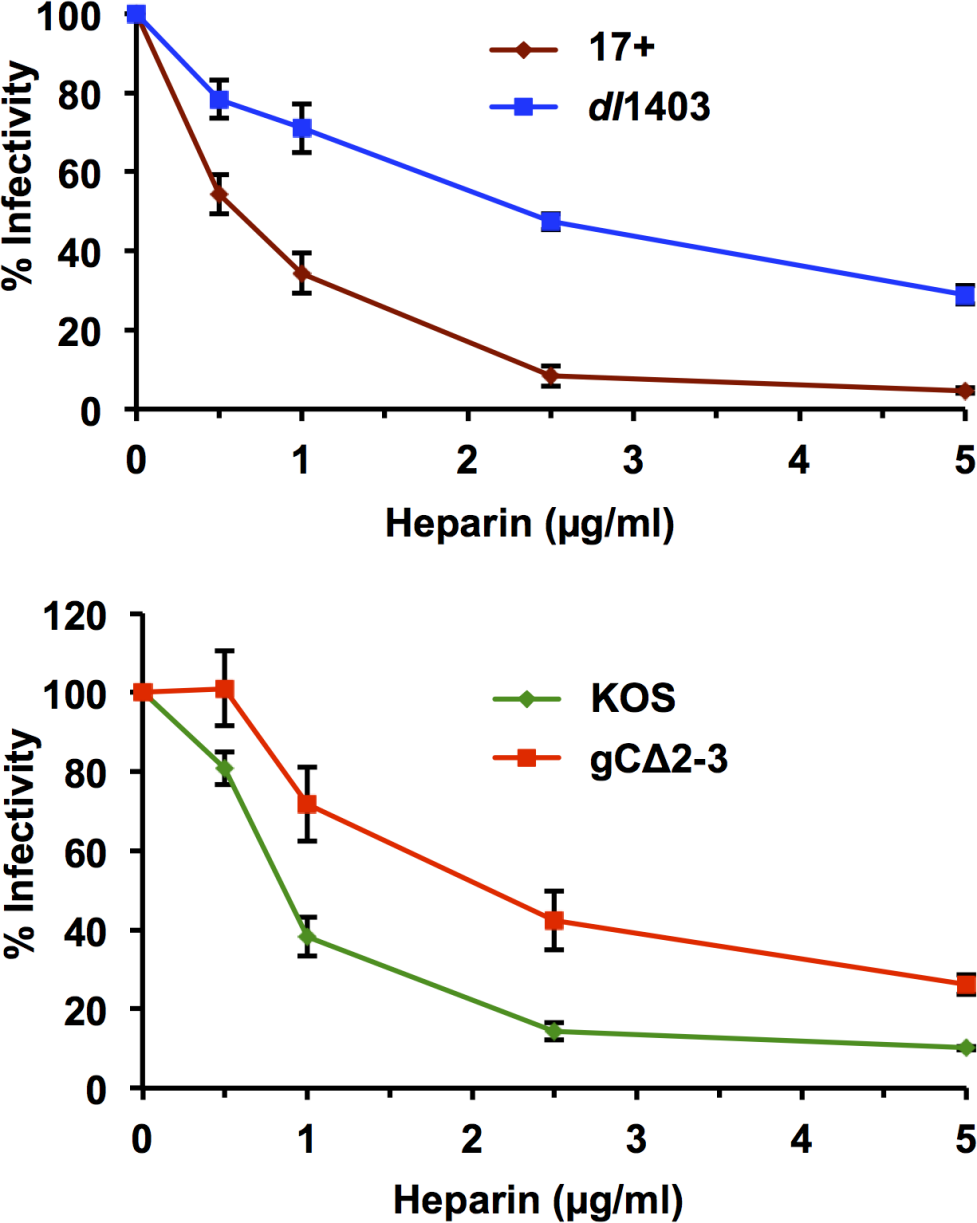
Resistance of HSV-1 *dl*1403 to inhibition by heparin. HSV-1 17+, *dl*1403, KOS, or gC∆2–3 (100 PFU per well) was added to Vero cells at 4˚C for 1 hr in the presence of 0 to 5 μg/ml heparin as indicated. Cultures were washed thrice with PBS, and then incubated at 37˚C for 24 hr. Plaque formation was detected by immunoperoxidase staining. Plaque formation in the untreated sample was set to 100%. The data are means of quadruplicate determinations with the standard error of the mean. Results are representative of three independent experiments.

Envelope gC is the principal mediator of HSV-1 attachment to cell surface glycosaminoglycans, such as heparan sulfate [53,54,55]. In the absence of gC, gB mediates attachment of HSV-1 to heparan sulfate proteoglycans [46]. We assessed the ability of heparin to inhibit the infectivity of *dl*1403. HSV-1 *dl*1403 was inhibited by heparin to a lesser extent than the gC-containing parental virus 17+ (Fig 5). Likewise, the infectivity of gC-null HSV-1 gC∆2–3 was inhibited to a lesser extent than its parental wild type virus, KOS (Fig 5), consistent with previous reports [56]. The results from Fig 5 suggest that *dl*1403 behaves similarly to a gC-null virus in an assay of biological function.

### HSV-1 ICP0-null mutants grow to similar titers regardless of gC expression

**Fig 6:**
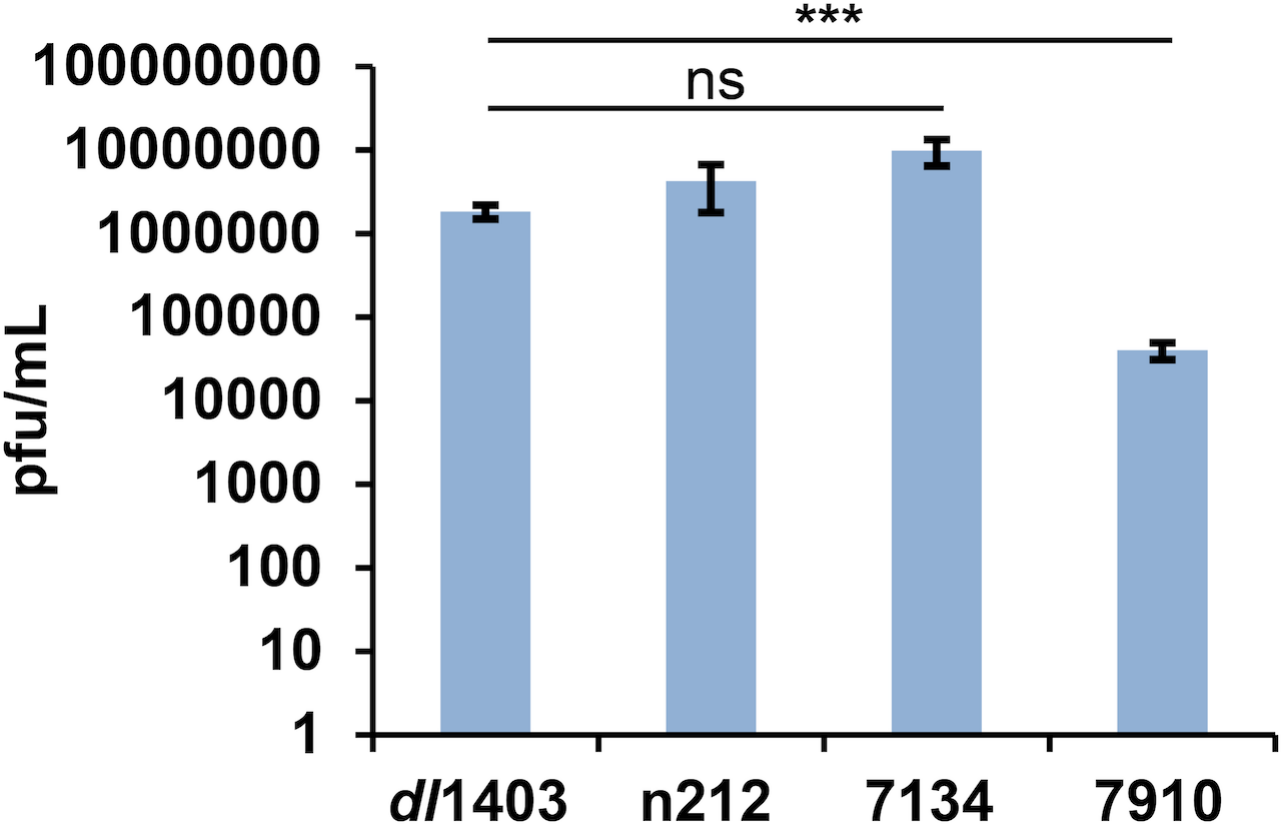
Loss of gC does not render HSV-1 *dl*1403 more attenuated than other ICP0-null strains with intact gC expression. HEL cells were infected with HSV-1 *dl*1403, n212, 7134, or 7910 for 24 hr (MOI of 10). Cells and supernatant were harvested, freeze-thawed three times, and then titered on U2OS cells in the presence of hexamethylene bisacetamide (HMBA). The data is the average of 3–4 independent replicates with the standard error of the mean. Statistical analysis was performed using one-way ANOVA and Tukey’s multiple comparison post-test. *** p <0.001, ns = not significant.

Upon the initial generation of *dl*1403, marker rescue experiments showed that restoration of the ICP0 sequence returned viral replication to wild type levels in cultured cells [4]. If the loss of gC had affected viral growth in culture, a decrease in the growth of the rescued virus would have been expected, suggesting that in this background in cultured cells, the additional disruption of gC does not decrease the infectivity of *dl*1403. Similarly, when grown on HEL fibroblasts and titered on U2OS cells, as previously described [12], we found that *dl*1403, n212 and 7134 all reached similar titers (Fig 6). This suggests that despite the loss of gC, *dl*1403 is not more attenuated than other ICP0-null viruses with intact gC expression. Interestingly, all three of these viruses reach higher titers than what is observed for 7910, which may be a strain-specific difference.

In summary, HSV-1 *dl*1403 virions and strains derived from it, such as D8 and FXE, lack gC, and *dl*1403-infected cells fail to synthesize gC, likely due to detected mutations in the gC gene. Our results illustrate the potential for secondary mutations in the construction and evaluation of HSV-1 mutant strains. In general, it is desirable to construct and examine the same mutations in multiple wild type strains. Although our stocks of the parental 17+ strain lack the gC mutations, it is possible that the stock of 17+ from which *dl*1403 was originally derived was the original source of the mutations. Notably, laboratory strains of HSV-1 considered to be wild type also contain mutations in their genomes [57,58,59,60,61].

Several strains of HSV-1 that lack gC have been identified [62,63,64,65]. These strains are viable in cell culture, consistent with the non-essential role of gC in HSV-1 replication. While HSV-1 gC plays a well-defined role in virion attachment to cell surface glycosaminoglycans [66], it is not an absolute requirement for HSV-1 entry via endocytic and non-endocytic pathways [67,68]. Repair of the ICP0 deletion in HSV-1 *dl*1403 restores the virus to wild type phenotype in cell culture assays that test ICP0 functions [4,41]. For example, exogenous expression of ICP0 restores plaque formation efficiency of *dl*1403 to wild type in cell culture [4], suggesting that the observed phenotype of this mutant likely results from the loss of ICP0 and not gC. Studies using *dl*1403 in mouse models have also effectively used an ICP0 rescuant as a control [6]. HSV-1 gC also binds to complement component C3b, providing protection from antibody-independent neutralization [69,70]. Although this function of gC would not be necessary in cell culture, the loss of gC would be expected to have a more significant impact on experiments performed *in vivo*. Indeed, in some animal models, gC-null viruses are highly attenuated [69,71,72,73]. However, in others, the lack of gC does not affect disease outcome [71,74,75,76,77]. A previous study comparing the growth of 17 syn, *dl*1403, and the marker rescue virus R4 in mice indicated that R4 replicated similarly to 17 syn, although it did show some evidence of mild attenuation compared to the wild type virus [6]. This suggests that while the lack of gC in *dl*1403 may have produced minor additional defects in replication *in vivo*, the effect of the loss of ICP0 is much greater. Along with the present study, this also highlights the importance of using rescuants as controls in experiments using mutant HSV-1 strains. Phenotypes of HSV-1 *dl*1403 attributable to the absence of gC, but unrelated to ICP0 function, such as enhanced resistance to heparin (Fig 5), are also expected to manifest in other *dl*1403-derived viruses including ICP0 rescuants.

HSV-1 *dl*1403, *dl*1403R, D8 and FXE are widely used viruses. Thus, our finding that these strains contain a secondary mutation in gC impacts a large number of studies. We are currently in the process of creating repaired versions of *dl*1403, D8 and FXE with intact gC expression. Our findings suggest that caution is necessary in using the current viruses and also suggest that previous studies should be interpreted with the absence of both the ICP0 and gC genes in mind.
